# Apparent ileal digestibility of Maillard reaction products in growing pigs

**DOI:** 10.1371/journal.pone.0199499

**Published:** 2018-07-05

**Authors:** Sergio Salazar-Villanea, Claire I. Butré, Peter A. Wierenga, Erik M. A. M. Bruininx, Harry Gruppen, Wouter H. Hendriks, Antonius F. B. van der Poel

**Affiliations:** 1 Wageningen Livestock Research, Wageningen, The Netherlands; 2 Animal Nutrition Group, Wageningen University & Research, Wageningen, The Netherlands; 3 Laboratory of Food Chemistry, Wageningen University & Research, Wageningen, The Netherlands; 4 Agrifirm Innovation Center, Royal Dutch Agrifirm Group, Apeldoorn, The Netherlands; Universitat de Lleida-IRBLLEIDA, SPAIN

## Abstract

The absorption of Maillard reaction products (**MRP**) from dietary origin has been linked to the occurrence of chronic diseases. The aim of the present study was to determine the effects of toasting time of rapeseed meal (**RSM**) and the processing method of the diets (pelleting and extrusion) that included RSM on the apparent ileal digestibility (**AID**) of total lysine, fructosyl-lysine (**FL**), carboxymethyl-lysine (**CML**), carboxyethyl-lysine (**CEL**), lanthionine (**LAN**) and lysinoalanine (**LAL**) in growing pigs. The study consisted of a 2×3 factorial design with toasting time of RSM (60, 120 min) and diet processing method (mash, pelleted, extruded) as factors. Fifty growing pigs were individually fed one of the experimental diets for 4.5 consecutive days. Following euthanasia, samples of digesta were collected from the terminal 1.5 m of the small intestine. Increasing the toasting time of RSM increased the contents of FL, CML and CEL, whereas the additional effects of the diet processing methods were relatively small. Lysinoalanine and lanthionine were not detected in the diets; therefore, digestibility of these compounds could not be determined. The contents of FL, CML and CEL in the ileal chyme were positively correlated to their contents in the diets. The AID of the MRP from thermally-treated RSM were overall low and were not related to their contents in the diets. The AID of FL ranged between -8.5 and 19.1%, whilst AID of CML and CEL ranged from -0.2 to 18.3 and 3.6 to 30%, respectively. In conclusion, thermal treatments have clear effects on the contents of MRP in the diets. These compounds have relatively low digestibility in growing pigs.

## Introduction

Maillard reaction products (**MRP**) are formed during thermal processing of feed ingredients and diets. This group of compounds results from the covalent bond formed between a free reactive -NH_2_ group of an amino acid and the carbonyl group of a reducing sugar [1]. In proteins, the ε-amino group of lysine reacts in the Maillard reaction, and in some cases arginine has also been suggested to be susceptible [2]. Crosslinks between amino acids (e.g. lysinoalanine [**LAL**] or lanthionine [**LAN**]), mediated through formation of dehydroalanine, occur during processing without reducing sugars involved. The absorbed MRP can promote oxidative stress and initiate inflammatory responses linked to atherosclerosis [3], although there are also reports on their positive antioxidant effects [4].

Increasing the toasting time of rapeseed meal (**RSM**) lead to a decrease of the total and reactive lysine contents [5], indicating the occurrence of Maillard reactions. Ingredients (e.g. RSM) with different extents of damage are mixed into diets and hydro-thermally reprocessed (e.g. pelleting or extrusion) in order to manufacture feeds. The feed production methods could lead to further formation of MRP. For example, extrusion of casein-based diets led to an increase of 9.4-fold in the carboxymethyl-lysine (**CML**) content of the diets [6]. Absorption of protein-bound MRP and crosslinked amino acids has been estimated previously using several indirect methods, such as measurements in blood, urine and/or faeces [6–10]. The estimation of absorption by these methods could be confounded by endogenous formation of MRP [11] and deposition in tissues [8,12]. Accurate determinations of the absorption of individual MRP, such as their ileal digestibility, are lacking.

In addition to the above, many of the studies dealing with absorption and metabolism of MRP have used rats as model animals for humans [6,9,13]. Pigs have been suggested as a better model animal than rats for digestion in humans due to their digestive anatomical and physiological similarity [14,15]. Rats might be able to digest protein sources that are poorly digestible by humans [15]. For example, the true ileal nitrogen digestibility of rapeseed proteins was 84% in humans [16], whilst it was 95% in rats [17]. Feeding pigs using the same rapeseed protein isolate resulted in values closer to the ones measured in humans (91%) [18].

The aim of the present study was to determine the effects of toasting time of the RSM and the diet processing method on the apparent ileal digestibility (**AID**) of total lysine, fructosyl-lysine (**FL**), CML, carboxyethyl-lysine (**CEL**), LAN and LAL in growing pigs. We hypothesize that higher contents of the MRP due to longer toasting times and harsher diet processing methods would lead to lower AID of MRP in growing pigs, linked to a lower AID of total lysine.

## Materials and methods

The Central Committee of Animal Experiments (The Netherlands) approved the use of experimental animals under the authorization number AVD260002015139. All animals were euthanized under sodium pentobarbital anesthesia, and all efforts were made to minimize animal suffering. The furosine, LAL and CML standards were obtained from Polypeptides (Strasbourg, France), whilst the rest of the standards (^13^C_6_,^15^N_2_-lysine, lysine, LAN) were obtained from Sigma-Aldrich (Steinheim, Germany).

### Production of the rapeseed meals and diets

The experiment consisted of a 2×3 factorial arrangement, in which the factors were toasting time of the rapeseed meal (60, 120 min) and diet processing method (mash, pelleting, extrusion). This design resulted in 6 experimental diets: 60 min mash/pelleted/extruded (**M-60**, **P-60** and **E-60**, respectively) and 120 min mash/pelleted/extruded (**M-120**, **P-120** and **E-120**, respectively).

The experimental diets were produced according to the procedure described by Salazar-Villanea et al. [19]. The diets that included the untoasted RSM were excluded from this experiment due to the their low contents of MRP, LAN and LAL. Rapeseed meal was the only protein containing ingredient in the diets (Table 1) and titanium dioxide (3 g/kg) was added to all diets for the calculation of digestibility.

**Table 1:** Ingredient composition of the experimental diets.

Ingredient	Inclusion level (g/kg as is)
**Rapeseed meal**	410.0
**Potato starch ^a^**	503.9
**Soy oil**	60.0
**Monocalcium phosphate**	8.1
**Calcium carbonate**	5.5
**Premix vitamins and minerals ^b^**	5.0
**Titanium dioxide**	3.0
**Salt**	3.0
**Sodium bicarbonate**	1.5

^a^ Paselli™ (Avebe, Veendam, The Netherlands).

^b^ Composition premix per kg of feed: 100 mg Fe (as FeSO_4_·H_2_O), 70 mg Zn (as ZnSO_4_·H_2_O), 20 mg Cu (as CuSO_4_·5H_2_O), 30 mg Mn (as MnO), 1.2 mg I (as KI), 0.25 mg Se (as Na_2_SeO_3_), 125 mg antioxidant, 10000 IU Vit. A, 2000 IU Vit. D_3_, 150 mg choline chloride, 40 mg Vit. E, 30 mg niacine, 15 mg D-pantothenic acid, 4 mg Vit. B_2_, 1.5 mg Vit. K_3_, 1.5 mg Vit. B_6_, 1.0 mg Vit. B_1_, 0.4 mg folic acid, 0.05 mg biotine, 20 μgVit. B_12_. Carrier was potato starch (Paselli).

### Animals, feeding and housing

The experiment was performed with 50 crossbred boars (Pietrain×Topigs 20) with a starting body weight of 20.4 ± 0.9 kg. The source of the used pigs was Proefboerderij Laverdonk, Heeswijk-Dinther, The Netherlands. Pigs were housed in groups, but separated and individually fed twice per day (0800 and 1600 h) and remained separated for 1 h. After feeding, the pigs were re-grouped into their original groups. Pigs had free access to water during group and individual housing. Temperature in the experimental barn was 28 ± 1°C and the lights were turned off 12 h per day. The pens (0.71 m^2^/animal) had a 3:1 ratio of solid to slatted floor.

Each trial period consisted of 10 adaptation days, during which the animals were fed 30% test diet mixed with a commercial growing pig diet for the first 3 days and incremental amounts of the test diet (10% extra per day) until 100% was achieved on the 10^th^ day. In total, the animals were fed 100% test diet during 4.5 consecutive days. Pigs were fed 4 h before euthanasia on the morning of the 14^th^ day. The animals were fed at 2.8 × maintenance energy requirement (239 kJ NE/kg BW^0.75^; CVB, 2011), based on the measured body weight on days 8 and 13. Water (1:1.5 feed to water) was added before feeding to the mash diets to reduce dustiness of the diet. One animal from the M-120 diet was excluded before the end of the experiment due to low feed intake.

Euthanasia was performed by injection of pentobarbital in the ear vein and exsanguination. Following exsanguination, the small intestine was removed and 1.5 m anterior to the ileo-cecal valve were dissected. The contents from this section were flushed out with 50 ml demineralized water, immediately frozen in dry-ice and kept at -20°C until freeze-drying. After freeze-drying, the samples were ground through a 1 mm sieve using a centrifugal mill at 12000 rpm (ZM200, Retsch, Haan, Germany).

### Chemical analyses

Dry matter content was determined according to ISO 6496 (ISO 1999). Nitrogen content was analyzed by combustion (AOAC 1969) (Thermo Quest NA 2100 Nitrogen and Protein Analyzer; Breda, The Netherlands) and the crude protein (**CP**) content calculated using a 6.25 conversion factor. Titanium dioxide was determined by UV-spectroscopy as described before [20].

The contents of furosine, CML, LAL, LAN, CEL and Lys in the RSM, diets and ileal samples were quantified by UHPLC-MS. The samples (10 mg) were hydrolyzed with 1 ml 6 M HCl during 24 h at 110°C. The tubes were dried under N_2_ flow and the dried material was re-suspended in 1 ml UPLC-grade Milli-Q water, sonicated and centrifuged (16100 × g, 3.5 min, room temperature). The samples were kept at -20°C until analysis.

To test for ionisation efficiency, the recovery of a spike was measured by adding a known concentration of all standards to the supernatant of 18 randomly selected samples (after diluting the samples 10 or 50 times), using ^13^C_6_^15^N_2_-lysine as internal standard. Ionisation efficiency was calculated as the difference between the peak area of the spiked and non-spiked samples, divided by the standard injection alone, after correction of all values by the value for the internal standard. Average efficiencies for the samples diluted 50 times were 103, 103, 103, 103, 117 and 128% for furosine, CML, CEL, LAL, lysine and LAN. Corresponding average efficiencies for the samples diluted 10 times were 112, 81, 93, 85, 113 and 53%. As the effect of the sample matrix seemed stronger in the 10 times diluted samples, it was decided to use the samples diluted 50 times for quantification.

The supernatant of all samples was diluted 50 times in eluent A that contained 1 mg/l (w/v) ^13^C_6_,^15^N_2_-lysine as internal standard and centrifuged prior to injection. Eluent A was UPLC-grade Millipore water containing 0.1% (v/v) formic acid and eluent B was acetonitrile containing 0.1% (v/v) formic acid. The samples were analyzed using an Acella RP-UHPLC system (Thermo Scientific, San Jose, CA, USA) with an Acquity BEH Amide Vanguard precolumn (2.1 × 50 mm, 1.7 μm particle size) and an Acquity UPLC BEH 300 Amide column (2.1 × 150 mm, 1.7 μm particle size). The column was maintained at 35°C and the injection volume was 1 μl. The elution profile was as follows: 0–2 min isocratic on 80% B, 2–3 min linear gradient from 80% B to 65% B, 3–5 min isocratic on 65% B, 5–7 min linear gradient from 65% B to 40% B, 7–10 min isocratic on 40% B, 10–12 min linear gradient from 40% B to 80% B and 12–28 min isocratic on 80% B. The flow rate was 350 μl/min. Mass spectrometric data were obtained using an LTQ-VelosPro (Thermo Scientific) equipped with a heated ESI probe, coupled to the UHPLC system. The capillary voltage was set to 3 kV. The sheath gas flow rate was set at 20 and the auxiliary gas flow rate at 5 (arbitrary units). A selected reaction monitoring (SRM) method (Table 2) was used in negative ion mode for LAL and LAN and in positive ion mode for the other compounds. The normalized collision energy was set at 30 for furosine, Lys and LAL and at 35 for the other compounds and the m/z width on the parent fragment was set to 1. An external standard calibration curve with concentrations of 10, 5, 2.5, 1, 0.1 and 0.01 mg/l of each standard was used to calculate the content of each compound. Compounds were quantified using the external standard calibration curve by plotting MS peak area divided by the MS peak area of the labelled Lys, used as internal standard. The coefficient of variation in the intensity of the internal standard was 15.2%. Data were acquired and analyzed using XCalibur 2.2 software (Thermo Scientific). The quantification limits were 0.05 g/kg for LAN, 0.005 g/kg for LAL, 0.0005 g/kg for CML and CEL and 0.0004 g/kg for furosine and lysine.

**Table 2:** Selected reaction monitoring conditions.

Compound	Parent mass (Da)	Fragment (m/z)
**Lysine**	146	130
**^13^C_6_,^15^N_2_-Lysine**	154	137
**Carboxymethyl-lysine**	204	84, 130
**Lanthionine**	208	120
**Carboxyethyl-lysine**	218	84, 130
**Lysinoalanine**	233	128, 145
**Furosine**	255	84, 130

### Calculations and statistical analysis

Furosine was analysed in the present experiment as an indirect measurement for FL [10]. Furosine is produced after hydrolysis with 6M HCl at 110°C for 23 hours, where FL is degraded into 30.0 ± 1.2% furosine [21]. It is assumed in this study that the same reaction occurs with protein-bound FL originating from RSM. The content of FL in the diets and the ileal chyme was calculated by multiplying the determined furosine content by 3.3, based on the conversion of FL into furosine during acid hydrolysis [21]. These contents were used for the calculation of the AID of FL.

The AID was calculated using Eq 1:

$$AID\;of\;x\left(\%\right)=100-\left[\left(\frac{x_{chyme}}{x_{diet}}\right)\times \left(\frac{TiO_{2\;diet}}{TiO_{2\;chyme}}\right)\times 100\right]$$ (1)

were *x* represents the concentration of the compound (g/kg DM) in the chyme and in the diet, and TiO_2_ represents the concentration of the marker (g/kg DM) in the chyme and in the diet.

Effects of experimental treatments were tested by restricted maximum likelihood using the MIXED procedure of SAS (version 9.3) by means of an F-test according to a model with toasting time of RSM (**TT**), type of diet processing (**DP**) and their interaction as fixed factors. Feed intake of each of the animals on the day of euthanasia corrected for the average intake per treatment was used as covariate in the model. The CORR procedure of SAS was used to estimate correlations between MRP contents in the diets, the ileal chyme and their AID. Significance was assumed at *P*-values lower than 0.05, whilst trends were considered at *P*-values between 0.05 and 0.1. Post-hoc comparison between treatment means was performed using the Bonferroni adjustment.

## Results and discussion

### MRP content of the diets and the ileal chyme

The analysed MRP composition of the rapeseed meals and the diets are reported in Table 3. LAL and LAN concentrations in the diets were below the limit of quantification. Therefore, these were excluded from the digestibility calculations. Increasing the toasting time of RSM from 60 to 120 min, decreased the content of total lysine by 9% and increased the contents of FL, CML and CEL by 20, 60 and 45%, respectively. Assuming that 56% of the FL reverts back into lysine after hydrolysis with 6M HCl [21], 5 and 7% of the total lysine measured in the 60 and 120 min toasted RSM, respectively, can be considered as reverted lysine. Hence, the total loss of lysine after toasting accounts 14–16%. Comparing the 60 to the 120 min toasted RSM in the present study, the sum of the amounts of FL, CML and CEL formed, only accounts for 25% of the decrease in total lysine content. Similar results have been reported previously (Nguyen et al. 2016), as the sum of the Amadori products (calculated from measured furosine), CML and CEL after heating sodium caseinate suspensions with glucose or lactose (molar ratio 10:1) at 120 and 130°C for 30 min only represented approximately 21% of the total lysine loss. The remainder of the lysine could probably have reacted to other MRP products that were not analysed in the present study, for example 5-hydroxymethyl-2-furaldehyde (HMF), pyrraline or melanoidins [22,23]. Autoclaving of canola meal at 130°C for 20, 30 or 45 min has been previously reported to decrease the total lysine content, whilst increasing the content of FL (determined as furosine) [24].

**Table 3:** Chemically analyzed composition of the RSM and the experimental diets ^a^.

		Content (g/kg DM)						
Ingredient/diet	DM (g/kg as is)	CP	TiO_2_	Total lysine	Furosine	FL ^b^	CML	CEL
***Toasting time RSM***								
**60 min**	925.1	396.1	-	17.1	0.490	1.6	0.074	0.064
**120 min**	924.3	395.3	-	15.5	0.588	2.0	0.118	0.093
***Experimental diets***								
**60 min toasted RSM**								
**Mash**	916.6	155.8	2.2	7.1	0.179	0.6	0.038	0.028
**Pelleted**	900.7	161.1	2.3	6.4	0.127	0.4	0.038	0.026
**Extruded**	911.2	161.0	2.3	7.0	0.115	0.4	0.048	0.034
**120 min toasted RSM**								
**Mash**	917.7	157.4	2.4	6.6	0.206	0.7	0.062	0.044
**Pelleted**	905.4	157.0	2.3	5.8	0.212	0.7	0.059	0.042
**Extruded**	909.7	157.7	2.4	6.6	0.141	0.5	0.060	0.058

^a^ Abbreviations: CP, crude protein; DM, dry matter; RSM, rapeseed meal; FL, fructoselysine; CML, carboxymethyl-lysine; CEL, carboxyethyl-lysine.

^b^ FL = Furosine (g/kg DM) × 3.3. Based on Krause et al. [21].

The additional effect of the diet processing methods on the content of MRP was relatively small compared to the effects of toasting time (Table 3). A decrease in the content of total lysine of approximately 10% was noticed after pelleting of the diets, but not after extrusion (Table 3). The decrease in the content of total lysine after pelleting was not reflected in higher contents of FL, CML or CEL of the pelleted diets. The average content of FL in the experimental diets decreased by 15 and 30% comparing the mash diets to the pelleted and extruded diets, respectively. The average content of CML in the 60 min toasted RSM diets increased by 26% after extrusioncompared to the mash and pelleted diets. No effects of pelleting or extrusion compared to the mash diets were detected on the diets containing 120 min toasted RSM. Pelleting did not affect the average content of CEL compared to the mash diet. However, there was an increase of 28% in the average content after extrusion compared to the mash diet. The conditions employed during extrusion (temperature, shear) could facilitate the conversion of early MRP (FL) into advanced MRP (CML, CEL), as reported before for extrusion of casein-based biscuits [6].

The contents of CML in the diets used in the present study are within the range of values reported for foods consumed by humans as part of a western style diet (0–0.424 g/kg) [25]. The lowest CML-containing diets in the present study (M-60 and P-60) had higher contents than cereals (0.025 g/kg food), the highest CML food category analysed in the western style diets [25]. The highest content of CML in the present study (M-120) was more than 2-fold higher than that of cereals.

**Fig 1:**
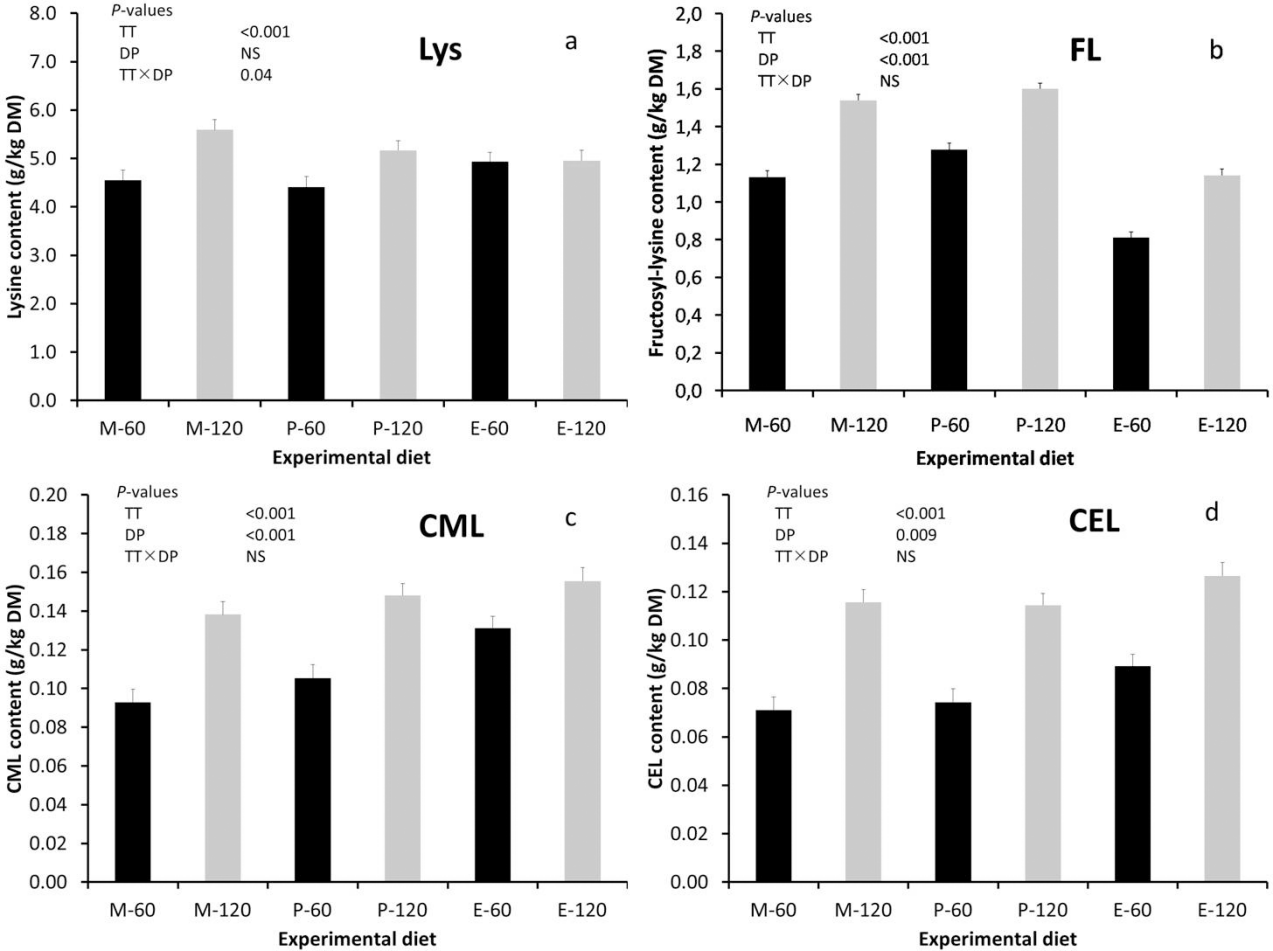
**Contents (g/kg DM) of (a) total lysine, (b) fructosyl-lysine, (c) carboxymethyl-lysine (CML) and (d) carboxyethyl-lysine (CEL) in the ileal chyme of RSM diets toasted for 60 or 120 min and either fed as mash, pelleted or extruded.** Means plus standard errors. Abbreviations: TT, toasting time; DP, diet processing; NS, not significant. Fructosyl-lysine (g/kg DM) = furosine (g/kg DM) × 3.3; based on Krause et al. [21].

There was no correlation (*P*>0.05) between the content of total lysine in the diets and that in the ileal chyme. The calculated content of reverted lysine in the chyme samples accounted for 10–15% of the total lysine content. The content of total lysine in the ileal chyme was affected (*P* = 0.04) by the interaction between toasting time and the diet processing method (Fig 1A). Whilst the total lysine content in the ileal chyme increased after extrusion of the 60 min toasted RSM compared to the mash and pelleted diets, in the 120 min toasted RSM diets there was a reduction after pelleting and an even further reduction after extrusion compared to the mash diets. The content of FL in the ileal chyme increased (*P*<0.001) with a higher toasting time, whereas it was reduced (*P*<0.001) after extrusion compared to the mash and pelleted treatments (Fig 1B). There was a positive correlation (r = 0.83, *P* = 0.04) between the content of FL in the diets and that in the ileal chyme. Significant effects (*P*<0.001) of toasting time and diet processing method were also obtained on the content of CML in the ileal chyme (Fig 1C). The average CML content in the ileal chyme was higher for the pigs fed the 120 min toasted RSM diets than for the pigs fed the 60 min toasted RSM diets (0.110 g/kg DM). It was also higher for those fed the extruded diets (0.143 g/kg DM) compared to the mash (0.116 g/kg DM) and pelleted (0.127 g/kg DM) diets. The CML content in the diets correlated positively (r = 0.93, *P* = 0.007) with that of the ileal chyme. Faecal excretion of CML has been reported to be proportional to the level of CML in the diets [6,8]. The ileal content of CEL (Fig 1D) was also affected by toasting time (*P*<0.001) and diet processing method (*P* = 0.009) and it was positively correlated (r = 0.98, *P*<0.001) to the CEL content of the diets. The significant positive correlations between the contents of MRP in the diets and the ileal chyme were likely due to the low and rather similar digestibility of the MRP analysed.

### Apparent ileal digestibility of MRP

The AID of total lysine decreased (*P*< 0.001) from 73.3% in the 60 min toasted RSM diets to 65.3% in the 120 min toasted RSM diets (Table 4). The AID of total lysine was negatively correlated to the content of FL in the diets (r = -0.82, *P*< 0.05) and tended to be negatively correlated to the CML content in the diets (r = -0.79, *P* = 0.06). Formation of MRP due to hydrothermal processing has been reported to have negative effects on lysine digestibility in growing pigs [24,26], which agrees with what was observed in the present study. The AID of total lysine in the mash diets (67.4%) was lower (*P*< 0.001) than that of the pelleted (68.9%) and extruded (72.0%) diets (Table 4). Pelleting and extrusion can drastically reduce particle size of the diets, fact that likely explains the increase in AID of total lysine [19,27]. In addition, it was shown previously that increasing the mechanical energy input during extrusion, resulting in higher shear, can decrease the size of the protein aggregates [28], which might facilitate enzymatic accessibility for protein hydrolysis. The AID of lysine results using the UHPLC-MS method in this study are similar to the our previous analyses using the amino acid analysis method [19] using the amino acid analysis method.

**Table 4:** Apparent ileal digestibility of total lysine, furosine, CML and CEL ^a^^,^ ^b^.

Experimental diet		Apparent ileal digestibility (%)			
	*n*	Total lysine	FL	CML	CEL
**60 min toasted RSM**					
**Mash**	8	73.0	19.1	10.6	17.2ab
**Pelleted**	8	72.8	-8.5	-0.2	6.0b
**Extruded**	9	74.0	18.6	10.9	21.3ab
**120 min toasted RSM**					
**Mash**	8	61.8	-1.2	10.8	3.6b
**Pelleted**	9	65.1	12.9	18.3	14.3ab
**Extruded**	8	70.0	0.2	13.9	30.0a
**SEM**		1.4	6.5	4.0	3.7
**P-values**					
**TT**		<0.001	0.33	0.05	0.74
**DP**		0.02	0.54	0.75	<0.001
**TT×DP**		0.10	0.01	0.10	0.01

^a^Abbreviations: n, number of animals; FL, fructosyl-lysine; CML, carboxymethyl-lysine; CEL, carboxyethyl-lysine; TT, toasting time; DP, diet processing method.

^b^ Different letters within a column per main effect represent significant differences between means (P<0.05).

An interaction effect (*P* = 0.01) between the toasting time and the diet processing method was observed for the AID of FL (Table 4). Although the effect of the interaction was significant for the AID of FL, there were no statistical pairwise differences between the AID of FL for the different diets. Previous studies [9], based on urinary excretion in rats, have suggested that only 3–5% FL is absorbed from the gastrointestinal tract of rats, in diets based on high levels of casein-bound FL (15.8 g/kg). Other studies [29] reported that only 16% of the FL intake in children fed a glucose-containing milk formula was excreted in urine. The values suggested by these studies [9,29] match the overall AID of FL values reported in the present study. In contrast, another study [30] reported an AID of FL of 46% (± 5.9%) for a heated casein-glucose mixture in growing pigs. The furosine content in that study (approximately 5.0 g/100 g crude protein), which was used to calculate FL, was more than 50-fold higher than the average furosine content of the diets in the present study (0.1 g/100 g crude protein). This could indicate that FL digestibility is related to its content in the diet. The negative AID of FL values for the P-60 and M-120 treatments (Table 4) could originate from endogenous FL production. Faecal excretion of FL has been reported in rats from control groups fed diets devoid of FL [9]. Alternatively, it has been suggested that the acidic environment in the stomach can revert up to 50% of the FL into lysine [11]. This could also contribute to the large variation in AID of FL reported in the present study. However, degradation of FL has only been demonstrated after harsh acid hydrolysis treatments (for example, 6 M HCl for 23 h at 110°C) [21]. Therefore, we consider that degradation of FL under gastric-like conditions is not very likely.

Toasting time tended (*P* = 0.05) to affect the AID of CML, which ranged from -0.2 to 18.3% (Table 4). Diet processing method or the interaction between toasting time and diet processing method did not affect the AID of CML. The urinary excretion of CML in rats fed various ingredients from dairy-origin ranged between 4 and 19% [31]. If the excretion reported by these authors is assumed to originate entirely from the diet and no endogenous production or organ retention is also assumed, these values match the absorption values reported in the present study. In other studies [6], urinary excretion of CML in rats was reported to reach 38.2% (± 12.2%) and 22.7% (± 6.5%) of the dietary intake of unextruded and extruded casein-based diets, respectively. Rats fed diets with high or low casein-bound CML contents were reported to excrete between 26 to 29% of their dietary intake in urine [9]. As in that study CML was not excreted in the urine of rats fed CML-free diets, all CML was assumed to be from dietary origin. We suggest that the AID of the several early and advanced MRP are related to the overall digestibility of the protein source. As protein from casein-based diets are more digestible than protein from the diets used in the present study (80–90% *vs* 60–75%, respectively), this might explain that the excretion of MRP (as a measurement of absorption) in those studies was higher than the absorption reported in our study.

The average AID of CEL ranged from 6.0 to 30.0% (Table 4) and was affected (*P*<0.01) by the interaction between toasting time and diet processing method. The AID of CEL decreased after pelleting of the 60 min toasted RSM diets, whilst it increased after extrusion. In contrast, the AID of CEL in the 120 min toasted RSM diets increased after pelleting and even further after extrusion. The increase in AID of CEL after extrusion of the 120 min toasted RSM diets could be related to the increase in CEL concentration in the diet. There are no previous reports on urinary/faecal excretion of CEL. The plasma level of CEL in rats fed bread crust was 25% higher compared to that of rats fed non-heated wheat starch diets [32].

In conclusion, AID of MRP from thermally-treated RSM in growing pigs was overall low and did not seem to be related to the content of MRP or processing method of the diets. Fructosyl-lysine and CML had a very limited digestibilities (-8.5 to 19.1%), whilst AID of CEL ranged from 3.6 to 30%, respectively. Considering these results and the similarities in digestive physiology between pigs and humans, we suggest that MRP would also have low digestibility in humans.
